# Complete Genome Sequence of *Bacillus* sp. Strain KICET-1, Isolated from Soybean Paste (Doenjang)

**DOI:** 10.1128/mra.00180-23

**Published:** 2023-05-31

**Authors:** Hyojung Park, Young-Ran Lee, Yeonsoo Kim, Jong In Won, Sun-A Lee, Byoung Seung Jeon

**Affiliations:** a Biomaterials and Processing Center, Korea Institute of Ceramic Engineering and Technology, Cheongju-si, Chungcheongbuk-do, Republic of Korea; b ReNhealbio, Daegu, Republic of Korea; University of Maryland School of Medicine

## Abstract

*Bacillus* sp. strain KICET-1, a bacterium isolated from traditional Korean soybean paste (Doenjang) at Osong, has one 4,099,652-bp DNA chromosome. The G+C content is 46.1%, and KICET-1 shares 99.64% similarity with Bacillus velezensis CR-502^T^ (AY603658), according to phylogenetic classification based on 16S rRNA gene sequences.

## ANNOUNCEMENT

Doenjang is a Korean fermented food that has dietary benefits (1). In this study, the whole genome of *Bacillus* sp. strain KICET-1, which was isolated from doenjang, was sequenced. KICET-1 was isolated by spreading doenjang samples on tryptic soy agar (TSA) containing skim milk, culturing the samples in an incubator at 30°C for 48 h, and selecting a red colony that created a clear zone. The 16S rRNA gene of KICET-1, as analyzed using the 27F and 1492R primers (2), was 99.64% consistent with that of Bacillus velezensis CR-502^T^.

To extract the genomic DNA (gDNA) of KICET-1, the isolate was cultured in tryptic soy broth (TSB) at 30°C for 48 h. The gDNA of KICET-1 was extracted with the Maxwell 16 tissue DNA purification kit (Promega, USA), and libraries were prepared using the TruSeq Nano DNA high-throughput library preparation kit (Illumina, USA). The gDNA was sequenced on the Pacific Biosciences (PacBio) Sequel system and Illumina HiSeq X Ten platform (151-bp paired-end reads) by Macrogen Inc. (Seoul, Republic of Korea). The gDNA was sheared using a Megaruptor 3 (Diagenode, USA) and, after library production, size selection with a cutoff value of <8 kb was performed using the BluePippin size selection system (Sage Science, USA). The PacBio Sequel production data were 181,296 reads and 1,707,593,753 bp. The *N*_50_ value for the long-read sequencing was 11,080 bp. After assembly was performed using subreads produced by the PacBio system, error correction was performed using reads produced by the Illumina system. Of the 15,761,780 reads (2,380,028,780 bp), 11,226,474 reads (1,693,537,068 bp) that had been subjected to filtering were used for analysis. Flye v.2.4.2 (3) was used to assemble subreads generated by the PacBio Sequel system. The reads in which 90% of the bases had Phred scores of ≥30 were used for error correction, and then adapter trimming was performed using Trimmomatic v.0.38 (options: ILLUMINACLIP:Adapter:fasta:2:30:10:8:true LEADING:15 TRAILING:15 SLIDINGWINDOW:4.15 MINLEN:36) (4). The assembly was polished with Pilon v.1.21 (5) in triplicate using high-quality adapter-trimmed Illumina reads. Preassembled read quality assessments were made possible by subread searches using the Basic Local Alignment Search Tool (BLAST) v.2.7.1 with the NCBI database. A circular map representing the contigs was created using Circos v.0.69.3 (6). The NCBI Prokaryotic Genome Annotation Pipeline (PGAP) v.6.4 (7) was used to perform gene predictions and functional annotations after the genome had been assembled. Default parameters were used for all software unless otherwise specified.

The *de novo* assembly generated a single chromosome with an estimated genome size of 4,099,652 bp and an average reference coverage of 375.8×. When the two ends of a contig overlap, the contig is regarded as a circular form. On the other hand, if there are no overlaps, then the contig might have been linear originally or there might be gaps at the end of the contig. As a result of Flye analysis, the genome was defined as one circular chromosome. The whole genome contained 4,124 identified genes, with 3,919 predicted to be coding sequences, 86 tRNAs, and 27 rRNAs. The G+C content was 46.1%.

### Data availability.

The whole-genome sequencing data for *Bacillus* sp. strain KICET-1 have been assigned GenBank accession number CP113515, 16S rRNA accession number OQ804551, BioProject accession number PRJNA906013, and BioSample accession number SAMN31888817, and the SRA numbers are SRR22729702 and SRR23314639. This strain has been deposited in the Korean Culture Center of Microorganisms (KCCM) with accession number KCCM 13284P.

## References

[1] Han DM, Chun BH, Kim HM, Jeon CO. 2021. Characterization and correlation of microbial communities and metabolite and volatile compounds in doenjang fermentation. Food Res Int 148:110645. doi:10.1016/j.foodres.2021.110645. 34507720

[2] Lane D. 1991. 16S/23S rRNA sequencing, p 115–147. *In* Stackebrandt E, Goodfellow M (ed), Nucleic acid techniques in bacterial systematics. John Wiley & Sons, Chichester, United Kingdom.

[3] Kolmogorov M, Yuan J, Lin Y, Pevzner PA. 2019. Assembly of long, error-prone reads using repeat graphs. Nat Biotechnol 37:540–546. doi:10.1038/s41587-019-0072-8. 30936562

[4] Bolger AM, Lohse M, Usadel B. 2014. Trimmomatic: a flexible trimmer for Illumina sequence data. Bioinformatics 30:2114–2120. doi:10.1093/bioinformatics/btu170. 24695404 PMC4103590

[5] Walker BJ, Abeel T, Shea T, Priest M, Abouelliel A, Sakthikumar S, Cuomo CA, Zeng Q, Wortman J, Young SK, Earl AM. 2014. Pilon: an integrated tool for comprehensive microbial variant detection and genome assembly improvement. PLoS One 9:e112963. doi:10.1371/journal.pone.0112963. 25409509 PMC4237348

[6] Krzywinski M, Schein J, Birol I, Connors J, Gascoyne R, Horsman D, Jones SJ, Marra MA. 2009. Circos: an information aesthetic for comparative genomics. Genome Res 19:1639–1645. doi:10.1101/gr.092759.109. 19541911 PMC2752132

[7] Tatusova T, DiCuccio M, Badretdin A, Chetvernin V, Nawrocki EP, Zaslavsky L, Lomsadze A, Pruitt KD, Borodovsky M, Ostell J. 2016. NCBI Prokaryotic Genome Annotation Pipeline. Nucleic Acids Res 44:6614–6624. doi:10.1093/nar/gkw569. 27342282 PMC5001611

