# Correction for Park et al., “Complete Genome Sequence of *Bacillus* sp. Strain KICET-1, Isolated From Soybean Paste (Doenjang)”

**DOI:** 10.1128/mra.00991-23

**Published:** 2023-12-01

**Authors:** Hyojung Park, Young-Ran Lee, Yeonsoo Kim, Jong In Won, Sun-A Lee, Byoung Seung Jeon

## AUTHOR CORRECTION

Volume 12, no. 6, e00180-23, 2023, https://doi.org/10.1128/mra.00180-23. Page 2, Acknowledgments, lines 3–7: “and the Technology…(MOTIE, Korea)” should read “and the National Research Foundation of Korea, NRF of the Republic of Korea (Development of technology for the production of 100% biomass-based bioplasticizers replacing petroleum-based plasticizers, grant no. 2022M3J4A1091415412).”

